# A287 HOME PARENTERAL NUTRITION FOR INDIVIDUALS WITH SHORT BOWEL SYNDROME SECONDARY TO CROHN’S DISEASE VERSUS OTHER ETIOLOGIES: A PROSPECTIVE COHORT STUDY

**DOI:** 10.1093/jcag/gwac036.287

**Published:** 2023-03-07

**Authors:** A N Sasson, J Noelting, K Schwenger, D Armstrong, M Raman, D Duerksen, S Whittaker, Y Lu, B Jurewitsch, L Gramlich, A Ananthakrishnan, J Allard

**Affiliations:** 1 Gastroenterology, University of Toronto, Toronto, Canada; 2 Gastroenterology, Essentia Health, Duluth, United States; 3 Gastroenterology, McMaster University, Hamilton; 4 Gastroenterology, University of Calgary, Calgary; 5 Gastroenterology, University of Manitoba, Winnipeg; 6 Gastroenterology, University of British Columbia, Vancouver; 7 Gastroenterology, McGill University, Montreal; 8 Pharmacy, St. Michaels Hospital, Toronto; 9 Gastroenterology, University of Alberta, Edmonton, Canada; 10 Gastroenterology, Massachusetts General Hospital, Boston, United States

## Abstract

**Background:**

Individuals with short bowel syndrome (SBS) have reduced intestinal absorptive capacity and many require home parenteral nutrition (PN) support. One of the common causes of SBS is secondary to intestinal resections in the management of Crohn’s disease (CD). Complication rates and survival in SBS secondary to CD on PN versus other etiologies remains unknown.

**Purpose:**

To determine whether patients with SBS secondary to CD versus SBS secondary to other etiologies on home PN, have increased risk of hospitalizations and complications including central line associated bloodstream infection (CLABSI) and venous thromboembolism and whether there is a difference in overall survival between groups.

**Method:**

This is a multicentre prospective cohort study using the Canadian Home Parenteral Nutrition (HPN) Registry on individuals with defined SBS separated into two cohorts: 1) Patients with SBS secondary to Crohn’s disease vs. 2) Patients with SBS secondary to other aetiologies (trauma, surgical complication, vascular event, volvulus, malignancy). Patient characteristics and clinical factors are presented as mean (standard deviation) for continuous variables and as frequency (percentage) for categorical variables. Comparison between groups (SBS CD vs SBS other) were performed using 2-sample t-test for continuous variables and Chi-square or Fisher exact tests when appropriate for categorical variable. Survival probabilities will be estimated using the Kaplan-Meier method.

**Result(s):**

The study included a total of 379 patients with short bowel syndrome on home PN. There are 170 (45%) patients with SBS secondary to CD and 209 (55%) patients with SBS from other secondary causes. The average age of those with CD is 52 and 65% female patients. The average age of those with other causes of SBS is 56 with similar percentage of female patients (65%). There were significant differences in baseline medications with higher use of immunosuppressant therapy (39% vs. 7%, p<0.001) in those with CD. There was no significant difference in total number of hospitalizations, hospitalizations related to PN and CLABSI.

**Image:**

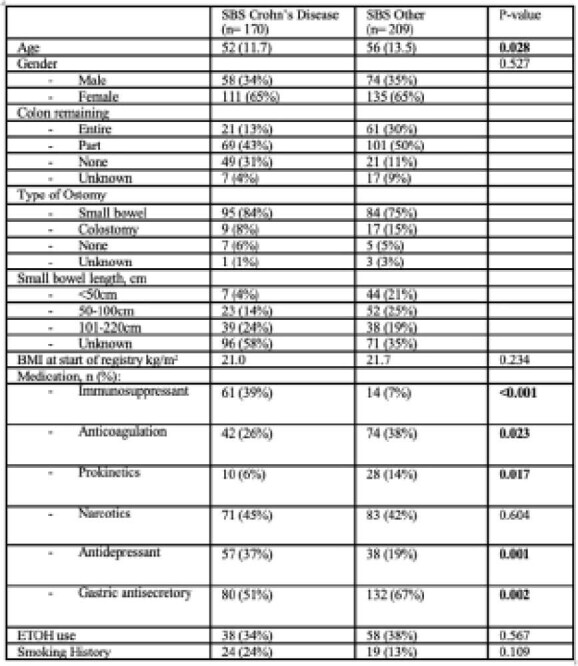

**Conclusion(s):**

Individuals with SBS secondary to CD do not appear to be at increased risk of central line infections or hospitalizations compared those with SBS from other causes.

**Please acknowledge all funding agencies by checking the applicable boxes below:**

None

**Disclosure of Interest:**

None Declared

